# Comparative injection-site pain and tolerability of subcutaneous serum-free formulation of interferonβ-1a versus subcutaneous interferonβ-1b: results of the randomized, multicenter, Phase IIIb REFORMS study

**DOI:** 10.1186/1471-2377-12-154

**Published:** 2012-12-06

**Authors:** Barry Singer, Daniel Bandari, Mark Cascione, Christopher LaGanke, John Huddlestone, Randy Bennett, Fernando Dangond

**Affiliations:** 1 Missouri Baptist Medical Center, St. Louis, MO, USA; 2 Multiple Sclerosis Center of Southern California and Research Group, Newport Beach, CA, USA; 3 Tampa Neurology Associates, South Tampa MS Center, Tampa, FL, USA; 4 North Central Neurology Associates, Cullman, AL, USA; 5 MultiCare Neuroscience Center of Washington, Tacoma, WA, USA; 6 EMD Serono, Inc., One Technology Place, Rockland, MA, USA

**Keywords:** Randomized controlled trial, Interferonβ-1a, Interferonβ-1b, Relapsing–remitting multiple sclerosis, Subcutaneous injections, Injection-site pain, Pain measurement

## Abstract

**Background:**

In patients with relapsing–remitting multiple sclerosis (RRMS), subcutaneous (sc) interferon (IFN)β-1a and IFNβ-1b have been shown to reduce relapse rates. A formulation of IFNβ-1a has been produced without fetal bovine serum and without human serum albumin as an excipient (not currently approved for use in the US). The objectives of this study were to evaluate tolerability, injection-site redness, subject-reported satisfaction with therapy, and clinical safety and efficacy of the serum-free formulation of IFNβ-1a versus IFNβ-1b in IFNβ-treatment-naïve patients with RRMS. The objectives of the extension phase were to evaluate long-term safety and tolerability of IFNβ-1a.

**Methods:**

This randomized, parallel-group, open-label study was conducted at 27 clinical sites in the US. Eligible patients aged 18–60 years were randomized to receive either IFNβ-1a, titrated to 44 μg sc three times weekly (tiw) (*n* = 65), or IFNβ-1b, titrated to 250 μg sc every other day (*n* = 64) over 12 weeks. Following this, all patients received IFNβ-1a 44 μg tiw for 82–112 weeks. Primary endpoint was mean change in patient-reported pain, as assessed by visual analog scale (VAS) diary pain score (from 0 mm [no pain] to 100 mm [worst possible pain]) at the injection site, from pre-injection to 30 min post-injection over the first 21 full-dose injections. Secondary assessments included proportion of patients pain-free as recorded by VAS diary and the Short-Form McGill Pain questionnaire VAS.

**Results:**

A total of 129 patients were included in the intent-to-treat analysis. Mean (standard deviation) change in VAS diary pain score was not significantly different between groups, although numerically lower with IFNβ-1a versus IFNβ-1b from pre-injection to immediately post-injection (1.46 [2.93] vs. 4.63 [10.57] mm), 10 min post-injection (0.70 [1.89] vs. 1.89 [5.75] mm), and 30 min post-injection (0.67 [2.32] vs. 1.14 [4.94] mm). Proportion of patients pain-free at all time periods post-injection was also not significantly different between groups. Adverse events were consistent with the known safety profiles of these treatments.

**Conclusions:**

In IFNβ-treatment-naïve patients with RRMS, both the serum-free formulation of IFNβ-1a and IFNβ-1b treatments were generally accompanied by low-level injection-site pain and were well tolerated.

**Trial registration:**

ClinicalTrials.gov NCT00428584

## Background

Clinical studies of subcutaneous (sc) interferon (IFN)β-1a and IFNβ-1b have shown that these disease-modifying drugs reduce relapse rates in patients with relapsing–remitting multiple sclerosis (RRMS) [1-4]. At the doses approved for the treatment of RRMS, both IFNβ-1a and IFNβ-1b have established long-term safety and tolerability profiles [5,6]. However, injections with these drugs are commonly associated with injection-site reactions (ISRs), injection-site pain, and flu-like symptoms (FLS), which can lead to poor adherence to treatment in some patients [7,8].

A formulation of IFNβ-1a has been developed without fetal bovine serum and without human serum albumin as an excipient, although this formulation is not currently approved for use within the US. In a 96-week study in patients with relapsing MS, the serum-free formulation of IFNβ-1a was associated with a lower prevalence of ISRs than had been seen in two earlier studies with the original IFNβ-1a formulation [9-11]. No randomized clinical study has yet compared the injection-site pain and tolerability profile of the serum-free formulation IFNβ-1a with that of another disease-modifying drug.

The primary objective of this study was to compare the tolerability of the serum-free formulation of IFNβ-1a, 44 μg sc three times weekly (tiw), with IFNβ-1b, 250 μg sc every other day (qod), as measured by the mean change in subject-reported injection-site pain from pre-injection to 30 min post-injection in IFNβ-treatment-naïve patients with RRMS during a 12-week period (comparative phase). During the extension phase, the primary objective was to evaluate long-term safety and tolerability of IFNβ-1a sc tiw.

Secondary efficacy endpoints included: the mean difference in injection-site pain from pre-injection to immediately post-injection and to 10 min post-injection, the proportion of pain-free patients, number and severity of relapses, assessments of the treatment of side effects, patient-rated treatment satisfaction, and rater-blinded assessment of injection-site redness.

Safety endpoints included analysis of adverse events (AEs), laboratory tests, physical examinations, vital signs, and concomitant medications.

## Methods

### Study design and patients

The Rebif New Formulation Versus Betaseron Tolerability Study (REFORMS) (ClinicalTrials.gov identifier: NCT00428584) was a randomized, multicenter, 2-arm, Phase IIIb study conducted at 27 clinical sites in the US. The study consisted of a 12-week randomized comparative phase, which was followed by a safety-extension phase of up to 112 weeks (range 82–112 weeks). The study was open-label, except for blinded assessments of ISRs. The initial central Institutional Review Board (IRB) submission was approved by Coast IRB, Colorado Springs, Colorado and, later, Schulman Associates IRB, Cincinnati, Ohio. For those sites that were not permitted to use a central IRB for study approval, submissions were made to the local IRB. This study was performed in accordance with the study protocol, the Declaration of Helsinki, the International Conference on Harmonization (ICH) Harmonized Tripartite Guideline for Good Clinical Practice (GCP), and all applicable regulatory requirements. Patients provided written informed consent for participation in the study.

Eligible patients were 18–60 years of age, had a primary diagnosis of RRMS as defined by the Poser or 2005 revised McDonald criteria [12,13], and had not previously received IFNβ treatment. Patients were not eligible if they had used any other approved disease-modifying treatment for MS (e.g. glatiramer acetate) or any cytokine or anti-cytokine treatment within 3 months before study initiation, used any immunomodulatory or immunosuppressive treatment within 12 months before study initiation, used any investigational drug or experimental procedure within 12 weeks before screening, received oral or systemic corticosteroids or adrenocorticotropic hormone within 30 days of study initiation, or used other injectable medications on a regular basis during the week before screening. Other exclusion criteria included having an alternative diagnosis to RRMS and being pregnant or breastfeeding. Women of childbearing potential were required to use appropriate contraception. All patients provided written informed consent.

### Treatments

**Figure 1:**
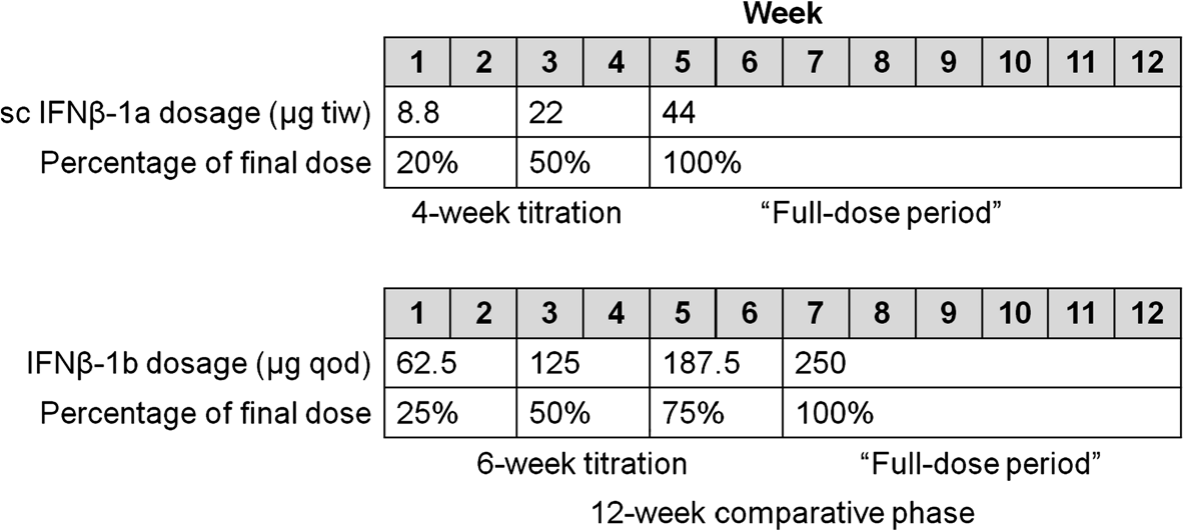
**Titration schedules for subcutaneous IFNβ-1a and IFNβ-1b.** The first 21 injections of full-dose IFNβ-1a and IFNβ-1b treatment were termed the “full-dose period”. IFN, interferon; qod, every other day; tiw, three times weekly.

Patients were randomized 1:1 to receive either the serum-free formulation of IFNβ-1a 44 μg sc tiw or IFNβ-1b 250 μg sc qod for the 12 weeks of the comparative phase. Treatments were allocated using a computer-generated randomization code. The doses of IFNβ-1a and IFNβ-1b were up-titrated at the beginning of the study according to the US prescribing information for each drug (Figure 1) [14,15]. Following the 12-week comparative phase, all patients received the serum-free formulation of IFNβ-1a 44 μg sc tiw during the safety-extension phase. Patients who transitioned from IFNβ-1b to IFNβ-1a could be up-titrated to the full dose of IFNβ-1a at the discretion of the investigator. Patients who did not wish to transition from IFNβ-1b to IFNβ-1a were withdrawn from the study. The length of the extension phase varied between 82 and 112 weeks, depending on the patient’s date of enrollment. The extension phase ended within 14 days of when the last enrolled patient completed the last visit at Week 94.

All patients self-administered IFNβ using the Rebiject II® autoinjector (EMD Serono, Inc., Rockland, MA, USA) with a 29-gauge needle for IFNβ-1a or Betaject® (with a 27-gauge needle), Betaject Lite® (with a 30-gauge needle), or Betaject® 3 (with a 27-gauge needle) (Bayer HealthCare Pharmaceuticals Inc., Montville, NJ, USA). Acetaminophen was given prophylactically at the discretion of the treating physician and dosed as needed to ameliorate constitutional symptoms (e.g. fever, myalgia, and FLS). Nonsteroidal anti-inflammatory drugs were given and dosed as needed at the discretion of the treating physician if acetaminophen failed to alleviate or prevent constitutional symptoms or if patients were allergic to, or unable to tolerate, acetaminophen.

### Assessments

Patient-reported pain was evaluated in a visual analog scale (VAS) diary and the Short-Form McGill Pain Questionnaire (SF-MPQ) [16]. Patients used the VAS diary to record the level of pain on a scale from 0 mm (no pain) to 100 mm (worst possible pain), immediately before, immediately after, 10 min after, and 30 min after the injection. The SF-MPQ also included a VAS for patients to record the level of the maximum amount of pain experienced during the 60 min after injection, from 0 mm (no pain) to 100 mm (worst possible pain). In addition, patients were requested to describe the types of pain that they experienced during the 60 min after injection. Patients completed the VAS diary and the SF-MPQ after every injection during the comparative phase and for the first 4 weeks of the safety-extension phase.

The Multiple Sclerosis Treatment Satisfaction Questionnaire (MSTSQ) adapted from Cramer *et al*. [17] included patient assessments of mood, treatment satisfaction, FLS, and ISRs. The MSTSQ was issued to patients at Weeks 2, 4, 6, 8, 12, 16, 24, 36, and 48. Mean values are reported for each treatment phase.

ISRs were assessed at each visit during the first 48 weeks by a healthcare professional who was blinded to treatment assignment. ISR measures included the diameter of injection-site redness, injection-site swelling, bruising, and consideration of patient-reported itching, within 72 h of the most recent injection.

Compliance was recorded throughout the study and was defined as the actual number of injections divided by the expected number of injections, expressed as a percentage. Safety assessments included AEs (coded to system organ class and preferred term using the MedDRA dictionary [Version 9.1] and summarized by severity and relationship), vital signs, hematology, and serum chemistry. Analgesic use among patients with and without AEs related to FLS was summarized by treatment group during the comparative phase and by treatment group and overall population during the extension phase.

The primary endpoint was the mean change in the VAS diary pain score from pre-injection to 30 min post-injection over the first 21 injections of full-dose IFNβ-1a and IFNβ-1b treatment (“full-dose period”). Due to the different titration schedules and dose frequencies of each treatment, the first 21 full-dose injections were administered during Weeks 5–11 in the IFNβ-1a group and during Weeks 7–12 in the IFNβ-1b group (Figure 1). Secondary endpoints included mean changes in the VAS diary pain score from pre-injection to immediately post-injection and 10 min post-injection; MSTSQ assessments; rater-blinded assessment of the mean diameter of injection-site redness; and SF-MPQ assessments, including the proportion of patients pain-free as recorded on the SF-MPQ VAS. Types of pain and severity experienced by the patient were also recorded on the SF-MPQ. The number of relapses and severity were secondary efficacy endpoints. Relapses were patient-reported and not objectively assessed; the number and severity of relapses were observational clinical assessments.

### Statistical analysis

The primary analysis population was the intent-to-treat population (all patients randomized to treatment). The safety population consisted of all patients who received at least one injection of study drug. The safety-extension population consisted of all patients who received at least one injection of study drug and had available extension phase data. Baseline characteristics of the two treatment groups were compared using analysis of variance (ANOVA) with effects for treatment group and pooled site for continuous variables and the Cochran–Mantel–Haenszel general association test, adjusted for pooled site, for categorical variables.

The null hypothesis was that there would be no difference in mean change in VAS pain score at 30 min post-injection from pre-injection across the treatments at full dose. The primary endpoint was evaluated with a two-way ANOVA model on signed ranked data, including treatment group and pooled site as main effects. The same method was also used to analyze treatment comparisons of the mean changes in the VAS diary pain score from pre-injection to immediately post-injection and 10 min post-injection, as well as mean SF-MPQ pain score at 60 min post-injection. An ANOVA model was used for between-group comparisons; for MSTQ scores, treatment group and pooled site were main effects; for injection-site redness, treatment group and site were main effects. The proportion of patients pain-free on SF-MPQ VAS was analyzed using the Cochran–Mantel–Haenszel general association test, adjusted for site, or Fisher’s exact test, if appropriate. Injection-site swelling, bruising, and itching were compared between groups using a Cochran–Armitage trend test. In the comparative phase, all statistical tests were two-sided and used a significance level of *α* = 0.05. No adjustment was made for multiple comparisons.

Patient-reported relapses during the comparative phase were compared using a Poisson regression model with the total number of relapses as the dependent variable and treatment group and pooled site as independent variables.

### Determination of sample size

A total of 100 patients (50 per arm) was calculated to provide at least 90% power to detect the difference between treatment groups for the primary objective, when the expected treatment effect size (the difference between the treatment groups divided by the standard deviation [SD]) was at least 0.735. The effect size was based on a difference between the treatment groups of 0.025 mm and an SD value of 0.034 mm. The difference between the treatment groups was based on a mean change of 0.1 mm in the IFNβ-1a group and 0.125 mm in the IFNβ-1b group, and assumed that the mean VAS diary pain scores at pre-injection in the two treatment groups were similar. The calculation also assumed a two-sided Wilcoxon rank sum test, a common SD of the change of ≤0.034 mm, and a Type I error rate of 5%.

## Results

### Patient disposition and baseline characteristics

**Figure 2:**
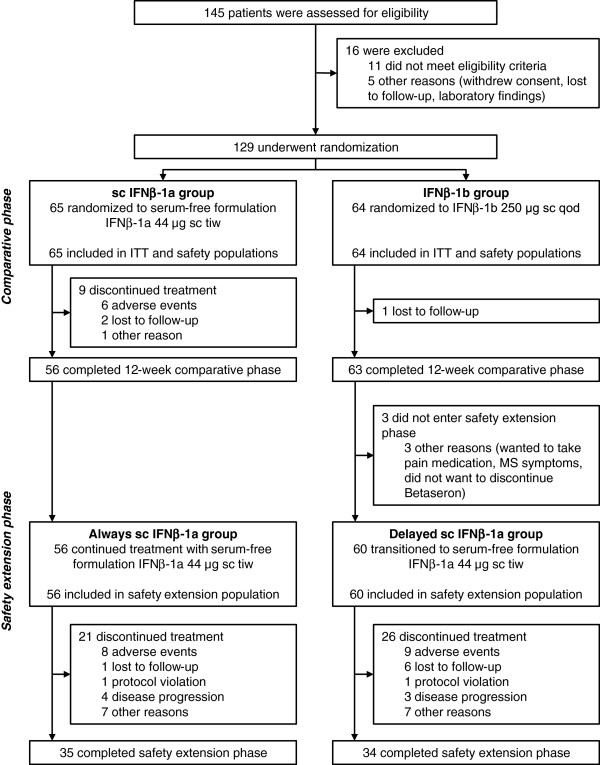
**Patient enrollment and disposition**. IFN, interferon; ITT, intent-to-treat; qod, every other day; sc, subcutaneous; tiw, three times weekly.

Between May 2006 and July 2009, a total of 129 patients were enrolled: 65 were randomized to IFNβ-1a and 64 to IFNβ-1b (Figure 2). Patient baseline characteristics (Table 1) did not differ significantly between groups. Fifty-six patients in the IFNβ-1a group completed the comparative phase and entered the safety-extension phase, and these were termed the “Always IFNβ-1a” group (Figure 2). Of the 63 patients in the IFNβ-1b group who completed the comparative phase, 60 entered the extension phase and were termed the “Delayed IFNβ-1a” group. During the extension phase, the mean (SD) duration of treatment with IFNβ-1a was longer in the Always IFNβ-1a group (436 [251] days) than in the Delayed IFNβ-1a group (338 [260] days).

**Table 1:** Baseline characteristics of patients (intent-to-treat population) randomized to receive subcutaneous IFNβ-1a or IFNβ-1b

**Baseline characteristic**	**IFNβ-1a (*N *= 65)**	**IFNβ-1b (*N *= 64)**
Age, years		
Mean (SD)	40.26 (9.80)	40.78 (9.56)
Median (range)	40.0 (20–60)	40.0 (19–59)
Female, *n* (%)	46 (70.8)	44 (68.8)
Race, *n* (%)		
White	55 (84.6)	58 (90.6)
Black	6 (9.2)	5 (7.8)
Asian	2 (3.1)	0
Other	2 (3.1)	1 (1.6)
BMI, kg/m^2^, mean (SD)	29.66 (6.76)	30.23 (8.35)
Classification of MS, *n* (%)		
Poser criteria	19 (29.2)	17 (26.6)
McDonald criteria	46 (70.8)	47 (73.4)
Time since first signs and/or symptoms of MS (onset), years, mean (SD)	4.51 (6.70)	5.74 (6.66)
Time since MS diagnosis, years, mean (SD)	1.01 (2.35)	1.93 (4.02)
Patients with no relapse during the 12 months before informed consent, *n* (%)	10 (15.4)	14 (21.9)
Relapses per patient,^a^ mean (SD)	1.36 (0.52)	1.30 (0.46)
Time since last relapse,^a^ months, mean (SD)	3.52 (2.94)	4.01 (2.93)
Number of steroid courses required for relapses per patient,^a^ mean (SD)	0.53 (0.60)	0.46 (0.50)
Patients who required ≥1 course of steroids,^a^*n* (%)	26 (47.3)	23 (46.0)

^a^Based on total number of patients with relapses during the 12 months before informed consent.

BMI, body mass index; IFN, interferon; MS, multiple sclerosis; SD, standard deviation.

### Tolerability

#### Comparative phase

**Figure 3:**
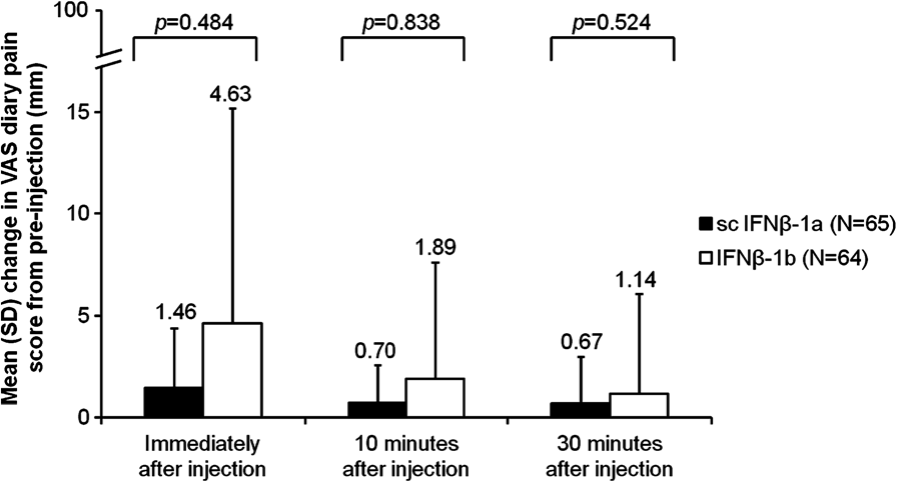
**Mean change in VAS diary pain score during full-dose treatment in the comparative phase (intent-to-treat population).** The VAS ranged from 0 mm (no pain) to 100 mm (worst possible pain). The mean change was calculated from the mean of 21 full-dose injections for each patient. IFN, interferon; SD, standard deviation; VAS, visual analog scale.

During the full-dose period, the VAS diary pain score was very low across both treatments. Mean changes in pain scores from pre-injection to immediately, 10 min, and 30 min after injection were all <5 mm with both treatments (Figure 3). The mean (SD) pre-injection VAS diary pain score was 0.43 (2.06) mm in the IFNβ-1a group and 0.40 (1.64) mm in the IFNβ-1b group. The primary endpoint of mean change in the VAS diary pain score from pre-injection to 30 min post-injection during the full-dose period was not statistically different between IFNβ-1a and IFNβ-1b (mean [SD] 0.67 [2.32] mm vs. 1.14 [4.94] mm, respectively, *p* = 0.524; Figure 3) but was numerically lower with IFNβ-1a than with IFNβ-1b. Mean changes in the VAS diary pain score from pre-injection to immediately and 10 min post-injection during the full-dose period were also not statistically different across groups (Figure 3).

**Figure 4:**
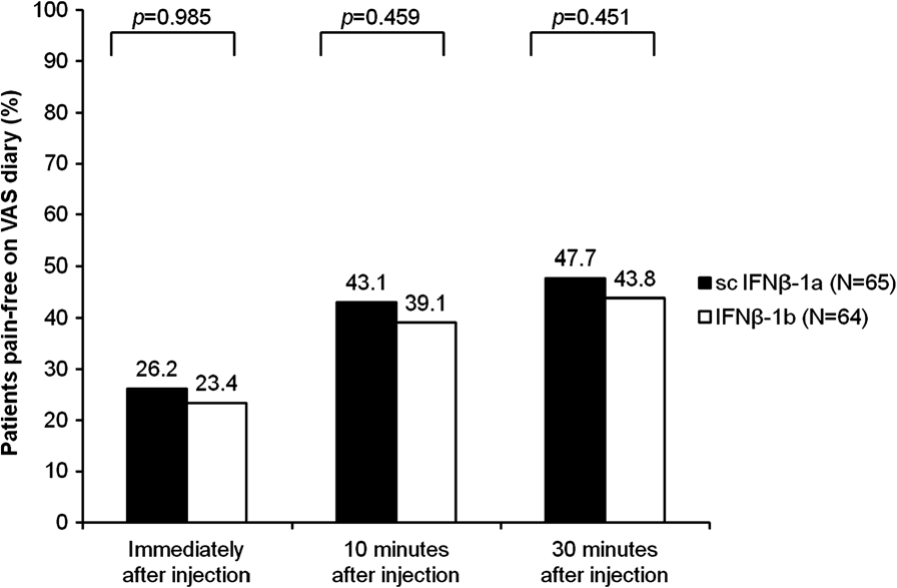
**Patients who reported as pain-free on the VAS diary in the comparative phase (intent-to-treat population).** Pain-free was defined as a VAS diary pain score of 0 mm (on a scale from 0 [no pain] to 100 mm [worst possible pain]) for all 21 full-dose injections. IFN, interferon; VAS, visual analog scale.

The proportions of patients who were pain-free on the VAS diary during the full-dose period (score of 0 mm for all full-dose injections) immediately, 10 min, and 30 min after injection were not statistically different across groups, but scores were numerically higher with IFNβ-1a than with IFNβ-1b (Figure 4). The mean SF-MPQ VAS pain scores were similar between the two groups, as were the proportions of patients who were pain-free on the SF-MPQ VAS (Table 2). During the full-dose period, the most common types of pain experienced during the 60 min after injection (incidence of ≥20% in either group) were hot-burning (reported by 40.0% of patients in the IFNβ-1a group vs. 53.1% of patients in the IFNβ-1b group), aching (29.2% vs. 45.3%), sharp (35.4% vs. 42.2%), tender (33.8% vs. 35.9%), shooting (26.2% vs. 34.4%), stabbing (29.2% vs. 32.8%), throbbing (27.7% vs. 32.8%), and heavy (9.2% vs. 23.4%).

**Table 2:** MSTSQ, ISR, and SF-MPQ endpoints during the full-dose period of the comparative phase (intent-to-treat population)

	**IFNβ-1a (*N *= 65)**	**IFNβ-1b (*N *= 64)**	***p*-value**
**MSTSQ assessments**			
MSTSQ overall satisfaction score,^a^ mean (SD)	1.51 (0.56)	1.53 (0.63)	0.616
MSTSQ injection system score,^a^ mean (SD)	1.68 (0.41)	1.80 (0.45)	0.156
MSTSQ score for background information,^a^ mean (SD)	2.28 (0.91)	2.28 (0.86)	0.734
**Blinded assessment of ISRs**			
Diameter of injection-site redness, mm, mean (SD)	11.32 (14.88)	11.75 (15.53)	0.986
Patients, *n* (%), with:			
Injection-site swelling	19 (29.2)	16 (25.0)	0.848
Injection-site bruising	21 (32.3)	7 (10.9)	0.019
Injection-site itching	7 (10.8)	6 (9.4)	0.366
**SF-MPQ assessments**			
SF-MPQ VAS pain score,^b^ mm, mean (SD)	2.54 (7.98)	3.24 (8.78)	0.612
Patients pain-free on SF-MPQ VAS,^b,c^*n* (%)	17 (26.2)	17 (26.6)	0.852

^a^On the MSTSQ, a lower score indicates a more favorable response to treatment. ^b^The SF-MPQ VAS recorded the maximum amount of pain experienced during the 60 min after injection, from 0 mm (no pain) to 100 mm (worst possible pain). ^c^Pain-free was defined as an SF-MPQ VAS score of 0 mm.

IFN, interferon; ISR, injection-site reaction; MSTSQ, Multiple Sclerosis Treatment Satisfaction Questionnaire; SD, standard deviation; SF-MPQ, Short-Form McGill Pain Questionnaire; VAS, visual analog scale.

The proportion of patients who reported any occurrence of FLS during the entire 12-week comparative phase on the MSTSQ was 84.6% with IFNβ-1a and 93.8% for IFNβ-1b; for the titration period, the occurrence of FLS was 75.4% and 87.5% with IFNβ-1a and IFNβ-1b, respectively. For the full-dose period, the FLS score was 84.6% and 76.6% with IFNβ-1a versus IFNβ-1b, respectively. The difference in frequency of FLS between IFNβ-1a (mean 3.55; SD 1.45) and IFNβ-1b (mean 2.78; SD 1.4) was significant (*p* = 0.003). The ratio of the percentage of patients reporting ISRs on the MSTQ during the full-dose period was similar to that of the FLS score. The difference in frequency of ISRs between IFNβ-1a (mean 3.72; SD 1.56) and IFNβ-1b (mean 2.92; SD 1.45) was significant (*p* = 0.005). The mean MSTSQ scores for overall satisfaction, injection system, and background information were similar between the two groups during the full-dose period (Table 2).

Blinded assessment of ISRs during the full-dose period found that the diameter of injection-site redness was similar between the two groups (Table 2). The proportions of patients with injection-site swelling and itching were similar between the two groups, but the incidence of injection-site swelling was significantly greater with IFNβ-1b than with IFNβ-1a during the uptitration period (28.1% vs. 18.5%, respectively; *p* = 0.042). Injection-site bruising was significantly more common with IFNβ-1a than with IFNβ-1b during the full-dose period of the comparative phase (32.3% vs. 10.9%, respectively; *p* = 0.019) (Table 2). However, most incidents were mild in severity.

#### Safety-extension phase

During the first 4 weeks of the safety-extension phase, the mean changes in VAS diary pain scores from before injection to all timepoints after injection were all <3 mm in both the Always and Delayed IFNβ-1a groups. More than half of all patients were pain-free on the VAS diary 10 min after injection. MSTSQ assessments for FLS, overall satisfaction, injection system background information, and ISRs were similar in the Always and Delayed groups, although blinded assessment of ISRs were numerically lower (7.46 mm) for the Delayed group than the Always group (10.79 mm). The mean MSTSQ, ISRs, and SF-MPQ during the safety-extension phase are summarized in Additional file 1: Table S1.

### Compliance and analgesic use

Treatment compliance during the comparative phase was high: 64/65 (98.5%) patients receiving IFNβ-1a and 62/64 (96.9%) patients receiving IFNβ-1b adhered to their medication schedule ≥90% of the time. Similarly, of the patients enrolled in the safety-extension phase, 102/116 (87.9%) were ≥90% compliant. Throughout the study, analgesics were used by the majority of patients. The percentage of patients using analgesics did not differ greatly between groups in either phase of the study; during the full-dose period of the safety population, analgesic use in the IFNβ-1a and IFNβ-1b was 61.5% and 57.8%, respectively, and mean total (SD) dose was 16,652 (16,941) mg and 12,862 (10,159) mg, respectively. Post-injection analgesic use for the treatment of an ISR was recorded by 1/64 patients in the IFNβ-1b group. During the full dose period, concomitant analgesic use for treatment of an ISR was recorded by 4/65 patients in the IFNβ-1a group and by 1/64 patients in the IFNβ-1b group. During the extension phase, analgesic use in the Always IFNβ-1a group and Delayed IFNβ-1b group was 80.4% and 78.3%, respectively.

The most commonly used (>3 individuals) analgesics during both study phases included ibuprofen, paracetamol, naproxen, and acetylsalicylic acid.

### Safety

#### AEs during the comparative phase

AEs reported in ≥5% of patients in either group during the comparative phase are shown in Table 3. AEs that were more common in the IFNβ-1a group than in the IFNβ-1b group included ISRs, nausea, increased alanine aminotransferase (ALT), increased serum ferritin, and abnormal liver-function test. AEs that were more common in the IFNβ-1b group than in the IFNβ-1a group included depression, fatigue, and dizziness. Most AEs occurring during the comparative phase were mild to moderate in severity. Severe AEs that occurred in at least one patient in the IFNβ-1a group were influenza-like illness (*n* = 2), back pain (*n* = 2), and headache (*n* = 2). Severe AEs that occurred in at least one patient in the IFNβ-1b group were back pain (*n* = 2) and headache (*n* = 2). During the comparative phase, six patients (all in the IFNβ-1a group) discontinued due to an AE. The AEs resulting in discontinuation were elevated liver function tests (*n* = 2) and elevated liver function tests plus the following: muscle cramps and spasms, chills, and headache (*n* = 1); elevated ferritin (*n* = 1); leukopenia and neutropenia plus ISR (*n* = 1); and pregnancy (*n* = 1).

**Table 3:** TEAEs reported by ≥5% of patients in either group during the comparative phase (safety population)

**Adverse event**	**Number of patients (%)**	
	**IFNβ-1a (*N *= 65)**	**IFNβ-1b (*N *= 64)**
Influenza-like illness	20 (30.8)	18 (28.1)
Headache	17 (26.2)	16 (25.0)
Injection-site reaction	18 (27.7)	9 (14.1)
Injection-site erythema	8 (12.3)	8 (12.5)
Depression	4 (6.2)	8 (12.5)
Fatigue	3 (4.6)	9 (14.1)
Urinary tract infection	7 (10.8)	5 (7.8)
Extremity pain	6 (9.2)	6 (9.4)
Nausea	7 (10.8)	3 (4.7)
Insomnia	5 (7.7)	5 (7.8)
Injection-site pain	4 (6.2)	5 (7.8)
Alanine aminotransferase increased	8 (12.3)	1 (1.6)
Back pain	4 (6.2)	4 (6.3)
Dizziness	2 (3.1)	6 (9.4)
Muscle spasms	5 (7.7)	3 (4.7)
Pain	4 (6.2)	3 (4.7)
Diarrhea	2 (3.1)	5 (7.8)
Chills	5 (7.7)	2 (3.1)
Influenza	5 (7.7)	2 (3.1)
Injection-site bruising	5 (7.7)	2 (3.1)
Serum ferritin increased	6 (9.2)	0
Liver-function test abnormal	5 (7.7)	0

IFN, interferon; TEAE, treatment-emergent adverse event.

Serious AEs were reported in two patients. One patient receiving IFNβ-1a had high ferritin levels and was subsequently diagnosed with grade 3, stage 2, chronic hepatitis; the investigator considered this event probably related to the study medication. One patient receiving IFNβ-1b had cholelithiasis, which required cholecystectomy; this event was considered unrelated to the study medication.

#### AEs during the safety-extension phase

Most AEs occurring during the extension phase were mild to moderate in severity. AEs occurring in >5% of patients are listed in Additional file 2: Table S2. Severe AEs that occurred in the Always IFNβ-1a group were non-study-related post-surgery pain (*n* = 1), tooth extraction pain (*n* = 1) and headache (*n* = 2). Severe AEs that occurred in at least one patient in the Delayed IFNβ-1a group were influenza-like illness (*n* = 2), and headache (*n* = 2). During the extension phase, 17 patients (eight patients in the Always IFNβ-1a group and nine patients in the Delayed IFNβ-1a group) discontinued due to an AE.

Serious AEs were reported in three patients in the Always IFNβ-1a group: one patient had an accidental overdose of IFNβ-1a, one patient developed cholecystitis (the same patient who had high ferritin and chronic hepatitis in the comparative phase), and one patient experienced vertigo. Serious AEs were reported in four patients in the Delayed IFNβ-1a group: one patient experienced intestinal obstruction, one patient experienced diverticulitis, one patient had an accidental overdose of study drug, and one patient had a hip fracture. Both cases of accidental overdose were due to misunderstanding of the dosing regimen. The case of cholecystitis was considered unlikely to be related to the study medication, and the other four serious AEs that occurred during the extension phase were considered to be unrelated to the study medication.

#### Other safety assessments

The mean values of hemoglobin, hematocrit, red blood cell counts, platelet counts, white blood cell counts, and alkaline phosphatase were within normal limits in each arm throughout the entire study. In the IFNβ-1a group, mean aspartate aminotransferase (AST) and ALT values become elevated above normal limits during the comparative phase. Mean AST (normal range 0–35 U/L) peaked at Week 8 (mean [SD] of 45.1 [40.4] U/L) but returned within normal limits in the Always IFNβ-1a group by Week 36. Mean ALT (normal range 4–36 U/L) also peaked at Week 8 (mean [SD] of 71.6 [104.4] U/L) and then declined, returning within normal limits in the Always IFNβ-1a group by Week 48. In the IFNβ-1b group, mean AST and ALT values initially increased slightly from baseline and then became steady during the comparative phase, but remained within normal limits. In the extension phase, after transitioning to IFNβ-1a, mean AST values remained within normal limits, while mean ALT values rose above normal limits, peaking at Week 24 (mean [SD] of 42.3 [27.2] U/L), but returned within normal limits by Week 48.

The normal ranges for ferritin varied between the laboratories where the assay was performed. Analysis of individual patient data showed that 26/65 (40.0%) patients in the IFNβ-1a group and 12/64 (18.8%) patients in the IFNβ-1b group had an elevated ferritin value at any time during the comparative phase, with ferritin levels considered to be transiently elevated in two patients receiving IFNβ-1a and three patients receiving IFNβ-1b. In the extension phase, 26/56 (46.4%) patients in the Always IFNβ-1a group and 17/60 (28.3%) patients in the Delayed IFNβ-1a group had elevated ferritin at any time. Among these patients, ferritin was considered transiently elevated in 13 patients in the Always IFNβ-1a group and six patients in the Delayed IFNβ-1a group.

#### Relapse rate during treatment

At 12 weeks in the comparative phase, in the IFNβ-1a group, eight patients (12.3%) reported one relapse, and one patient (1.5%) reported two relapses. In the IFNβ-1b group, seven patients (10.9%) reported one relapse. The mean (SD) number of relapses per patient was significantly higher in the IFNβ-1a group (0.15 [0.40]) versus the IFNβ-1b group (0.11 [0.31]; *p* < 0.001).

In the extension phase, 14 patients (25.0%) in the Always sc IFNβ-1a group had at least one relapse. Twelve patients (20.0%) in the Delayed sc IFNβ-1a group reported at least one relapse. The mean (SD) annualized number of relapses was 0.28 (0.63) in the Always IFNβ-1a group and 0.56 (1.57) in the Delayed IFNβ-1a group; the difference between groups was not statistically significant.

## Discussion

This study was the first randomized clinical trial to compare the injection-site pain and tolerability profile of the serum-free formulation of IFNβ-1a with that of another disease-modifying drug used in the treatment of MS. Patients were allowed to manage their pain with analgesic drugs. There was a tendency towards less pain being reported in patients in the IFNβ-1a group who did not use analgesics compared with the IFNβ-1b group. In patients who did use analgesics, the dose of analgesics for FLS and ISRs varied widely between patients over both study phases, although mean total dose tended to be higher in the IFNβ-1a group than in the IFNβ-1b and Delayed IFNβ-1b groups. Overall, in the first 21 full doses of IFNβ-1a and IFNβ-1b, mean increases in VAS pain scores from before injection to all timepoints after injection were not significantly different and small (<5 mm) in both groups, indicating that sc injections with both treatments were well tolerated for pain and likely contributed to high patient compliance in both treatment groups.

Over 96% of patients in either treatment group were ≥90% compliant with treatment during the comparative phase. Similarly, 102/116 (87.9%) patients were ≥90% compliant during the extension phase. The high rates of long-term compliance suggest good tolerability with both IFNβ-1a and IFNβ-1b, although compliance is usually greater in the clinical trial setting than in everyday clinical practice, prompting the need for long-term real-world-evidence studies.

Differences between the treatment groups were also not statistically significant for a number of other secondary assessments for pain. These included the mean increase in VAS pain score at all three post-injection timepoints, and the percentage of patients pain-free on both the VAS diary and the SF-MPQ. The MSTSQ overall satisfaction, injection system, and background information scores also did not differ significantly between the treatment groups during the full-dose period of the comparative phase. Overall, the results suggest that the use of the new formulation of IFNβ-1a with auto-injectors did not adversely affect tolerability outcomes and user satisfaction any more than the commercially available formulation of IFNβ-1b.

Rater-blinded assessment of ISRs were also not statistically different between the groups, except in injection-site bruising, which was significantly more common with IFNβ-1a than with IFNβ-1b during the full-dose period, and injection-site swelling, which was significantly more common with IFNβ-1b than with IFNβ-1a during uptitration. The observed differences in these two parameters could be due to the different natures of the two β-IFNs, the contents of the formulations, and mechanics of the auto-injector devices. Determination of the plausible explanation would require further studies. Overall, most patients (>80%) did not experience FLS or ISRs on the MSTSQ during the comparative phase. Data on which side effects within the definition of FLS caused patients the most concern would be useful endpoints to study in future studies.

While relapse rate at Week 12 of the comparative phase was significantly higher for IFNβ-1a than for IFNβ-1b, the clinical relevance of these subject-reported relapses remains unclear. Importantly, the study was not designed to compare efficacy objectively, so the results of those analyses should be interpreted with caution. However, overall treatment satisfaction did not differ between patients treated with IFNβ-1a and those treated with IFNβ-1b. Very low VAS pain scores from before injection to all timepoints were also reflected in the overall population of the safety-extension phase.

AEs reported during the comparative and safety-extension phases were consistent with the known safety profiles of IFNβ-1a and IFNβ-1b. Elevated serum ferritin may be a useful biomarker for monitoring responses to IFNβ treatment [18]; in this study, elevated serum ferritin levels were observed in both groups, and more commonly so in the IFNβ-1a group than in the IFNβ-1b group.

The high discontinuation rate observed during the extension phase may be due to the occurrence of AEs and/or the perceived commitment of remaining in the study, when there was availability of the original formulation commercially, and where a treatment switch was inevitable at study termination.

A number of previous studies compared the side-effect profile of the original IFNβ-1a with that of IFNβ-1b in patients with MS [8,19-21]. In a small, non-randomized exploratory study of 20 patients, mean increases in VAS pain scores from before injection to immediately, 10 min, and 60 min after injection were greater in patients receiving IFNβ-1a 44 μg sc tiw than in patients receiving IFNβ-1b 250 μg sc qod [19], although comparisons between groups were not tested for statistical significance. In a larger randomized study of 301 patients with RRMS, treatment with either the original formulation of IFNβ-1a 22 μg sc once a week or IFNβ-1b 250 μg sc qod did not show any significant differences between the treatment groups in the rates of FLS or skin reactions [20]. In an observational cohort study of 445 patients with RRMS, a greater percentage of patients receiving IFNβ-1b 250 μg sc qod were pain-free over 15 full-dose injections immediately, 30 min, and 60 min after injection compared with patients receiving the original IFNβ-1a 44 μg sc tiw [21]. However, the study was not randomized or controlled. Injection of IFNβ-1a at room temperature to reduce possible sensation of cold burning as per the manufacturer’s recommendations for use was also assumed. In another observational cohort study, the percentage of patients reporting an ISR did not differ significantly between those receiving the original IFNβ-1a and those receiving IFNβ-1b [8], lending evidence to a mild, variable tolerability between sc preparations of IFNβ-1b and IFNβ-1a.

The serum-free formulation of IFNβ-1a was evaluated with the aim of improving local tolerability to injection. An additional potential benefit is reduced immunogenicity; following independent observations in a 96-week study, a lower prevalence of neutralizing antibodies was observed in patients receiving the serum-free formulation of IFNβ-1a, as compared with patients treated with the original IFNβ-1a [9,10]. However, in this present study, no data were collected on the development of neutralizing antibodies to IFNβ-1a to allow comparison with the above study findings.

### Limitations of the study

Injection depth may affect pain and ISRs, although the various injection depth options that patients used were not recorded. This study mirrored general-practice use of auto-injectors, whereby patients choose their individual depth setting.

The utilization of VAS pain scores at smaller-ranging scales, for instance, from 0 to 10 mm, may have been more useful in obtaining a more sensitive picture of injection-site pain with these therapies. Also, except for inspection of injection-site redness, the study was not blinded. Lack of patient blinding may have influenced some of the patient-rated measures such as ratings of relapse, pain, and FLS. The clinical relevance of patient-reported relapse data is unclear and so, with the lack of objective assessment of relapses, results of those analyses should be interpreted with caution. In the statistical analyses of patient-rated measures, no adjustments for multiple comparisons were made. Although a limitation, no statistically significant differences across groups on any of the patient-rated assessments were observed, suggesting that this aspect of the study design was not a serious weakness of the study. However, to ensure certainty in any future analyses, the blinding of patients is recommended to reduce any potential of bias in the patient-rated measures.

Although the safety-extension phase was useful in gaining data on longer-term tolerability to IFNβ-1a, large dropouts and confounding variables such as treatment switch from a high dose of IFNβ-1b to IFNβ-1a did not permit systematic analyses from which valid interpretations and conclusions could be made.

## Conclusions

The results from this study in IFNβ-treatment-naïve patients with RRMS regarding tolerability and safety of the serum-free formulation of IFNβ-1a and IFNβ-1b demonstrate that these treatments are accompanied by comparable and low levels of injection-site pain.

## Competing interests

Barry Singer has received research support from Acorda, Biogen-Idec, EMD Serono, Genzyme and Roche, and has been a speaker and/or consultant for Acorda, Bayer, Biogen Idec, EMD Serono, Genentech, Genzyme, Pfizer, Novartis, and Teva.

Daniel Bandari has been a speaker and/or consultant for Acorda, Biogen Idec, EMD Serono, Pfizer, Novartis, and Teva Neuroscience.

Mark Cascione has received research support from Novartis, Teva, EMD Serono, Genzyme, Sanofi-Aventis, and Biogen Idec, and compensation for consulting and/or honoraria for speaking from Acorda, Bayer, EMD Serono, Novartis, Pfizer, and Teva Neuroscience.

Christopher LaGanke has received research support and has been a consultant and/or received honoraria from Novartis, Acorda, Biogen Idec, Tech Neuroscience, Bayer, EMD Serono, Pfizer, Sanofi-Aventis, Genzyme, and Opexa. Christopher LaGanke has also been a consultant and/or received honoraria from Questcor. John Huddlestone has received fees for speaking and/or consulting from Bayer, Biogen Idec, EMD Serono, Pfizer, and Teva. Randy Bennett and Fernando Dangond are employees of EMD Serono, Inc.

## Authors' contributions

BS, DB, MC, CLaG, JH, RB and FD all had equal involvement in the concept, design and conduct of this study and drafted/reviewed the content of the manuscript. All authors have reviewed, read and approved the final version of the manuscript for submission to BMC Neurology.

## Pre-publication history

The pre-publication history for this paper can be accessed here:

http://www.biomedcentral.com/1471-2377/12/154/prepub

## Supplementary Material

Additional file 1 **Table S1.** Mean MSTSQ, ISR, and SF-MPQ during the safety-extension phase. Click here for file

Additional file 2 **Table S2.** Adverse events reported by ≥5% of all patients during the safety-extension phase. Click here for file
