# Supplementary material for: Comparative injection-site pain and tolerability of subcutaneous serum-free formulation of interferonβ-1a versus subcutaneous interferonβ-1b: results of the randomized, multicenter, Phase IIIb REFORMS study
Source: BMC Neurol. 2012 Dec 6;12:154. doi: 10.1186/1471-2377-12-154 (PMC3541262; doi:10.1186/1471-2377-12-154)
Supplement: Additional file 1 — Table S1. Mean MSTSQ, ISR, and SF-MPQ during the safety-extension phase. [file 1471-2377-12-154-S1.docx]

**SUPPLEMENTARY TABLES**

**Table 1 Mean MSTSQ, ISR, and SF-MPQ during the safety-extension phase**

|  | **Always IFN β-1a  (*N* = 56)** | **Delayed IFN β-1a  (*N* = 60)** | **All patients  (*N* = 116)** |
| --- | --- | --- | --- |
| **MSTSQ assessments** | | | |
| MSTSQ overall satisfaction score,^a^ mean (SD) | 1.53 (0.56) | 1.60 (0.62) | 1.57 (0.59) |
| MSTSQ injection system score,^a^ mean (SD) | 1.69 (0.34) | 1.70 (0.35) | 1.69 (0.34) |
| MSTSQ score for background information,^a^ mean (SD) | 2.39 (0.91) | 2.40 (0.89) | 2.40 (0.89) |
| Patients reporting FLS at any time during the extension phase, *n* (%) | 50 (89.3) | 53 (88.3) | 103 (88.8) |
| Patients reporting ISRs at any time during the extension phase, *n* (%) | 53 (94.6) | 53 (88.3) | 106 (91.4) |
| **Blinded assessment of ISRs^b^** | | | |
| Diameter of injection-site redness, mm, mean (SD) | 10.79 (13.89) | 7.46 (10.57) | 9.10 (12.37) |
| Patients, *n* (%), with:  Injection-site swelling  Injection-site bruising  Injection-site itching | 18 (32.1)  15 (26.7)  7 (12.5) | 13 (21.6)  16 (26.7)  6 (10.1) | 31 (26.7)  31 (26.7)  13 (11.3) |
| **SF-MPQ assessments^c^** | | | |
| SF-MPQ VAS pain score,^d^ mm, mean (SD) | 2.57 (7.61) | 1.43 (4.86) | 1.98 (6.35) |
| Patients pain-free on SF-MPQ VAS,^d,e^ *n* (%) | 24 (42.9) | 20 (33.3) | 44 (37.9) |

^a^On the MSTSQ, a lower score indicates a more favorable response to treatment. ^b^Assessed during weeks 12–48. ^c^During the first four weeks of the safety-extension phase. ^d^The SF-MPQ VAS recorded the maximum amount of pain experienced during the 60 min after injection, from 0 mm (no pain) to 100 mm (worst possible pain). ^e^Pain-free was defined as an SF-MPQ VAS score of 0 mm.

FLS, flu-like symptoms; IFN, interferon; ISR, injection-site reaction; MSTSQ, Multiple Sclerosis Treatment Satisfaction Questionnaire; SD, standard deviation; SF-MPQ, Short-Form McGill Pain Questionnaire; VAS, visual analog scale.
