# Supplementary material for: Comparative injection-site pain and tolerability of subcutaneous serum-free formulation of interferonβ-1a versus subcutaneous interferonβ-1b: results of the randomized, multicenter, Phase IIIb REFORMS study
Source: BMC Neurol. 2012 Dec 6;12:154. doi: 10.1186/1471-2377-12-154 (PMC3541262; doi:10.1186/1471-2377-12-154)
Supplement: Additional file 2 — Table S2. Adverse events reported by ≥5% of all patients during the safety-extension phase. [file 1471-2377-12-154-S2.docx]

**SUPPLEMENTARY TABLES**

**Supplementary Table 2**

**Adverse events reported by ≥5% of all patients during the safety-extension phase**

| **Adverse event** | **Number of patients (%)** | | |
| --- | --- | --- | --- |
|  | **Always IFN β-1a  (*N* = 56)** | **Delayed IFN β-1a  (*N* = 60)** | **All patients  (*N* = 116)** |
| Upper respiratory tract infection | 10 (17.9) | 7 (11.7) | 17 (14.7) |
| Urinary tract infection | 9 (16.1) | 7 (11.7) | 16 (13.8) |
| Fatigue | 6 (10.7) | 10 (16.7) | 16 (13.8) |
| Headache | 5 (8.9) | 10 (16.7) | 15 (12.9) |
| Influenza-like illness | 5 (8.9) | 9 (15.0) | 14 (12.1) |
| Sinusitis | 6 (10.7) | 4 (6.7) | 10 (8.6) |
| Depression | 1 (1.8) | 9 (15.0) | 10 (8.6) |
| Injection-site reaction | 4 (7.1) | 5 (8.3) | 9 (7.8) |
| Arthralgia | 3 (5.4) | 6 (10.0) | 9 (7.8) |
| Muscle spasms | 1 (1.8) | 8 (13.3) | 9 (7.8) |
| Extremity pain | 5 (8.9) | 4 (6.7) | 9 (7.8) |
| Dizziness | 5 (8.9) | 3 (5.0) | 8 (6.9) |
| Influenza | 5 (8.9) | 2 (3.3) | 7 (6.0) |
| Injection-site erythema | 2 (3.6) | 4 (6.7) | 6 (5.2) |
| Pharyngitis streptococcal | 3 (5.4) | 3 (5.0) | 6 (5.2) |
| Fall | 5 (8.9) | 1 (1.7) | 6 (5.2) |
| Pharyngolaryngeal pain | 4 (7.1) | 2 (3.3) | 6 (5.2) |

IFN, interferon.
